# Supplementary figures and images for: Effect of miR-34a on the expression of clock and clock-controlled genes in DLD1 and Lovo human cancer cells with different backgrounds with respect to p53 functionality and 17β-estradiol-mediated regulation
Source: PLoS One. 2023 Oct 13;18(10):e0292880. doi: 10.1371/journal.pone.0292880 (PMC10575541; doi:10.1371/journal.pone.0292880)

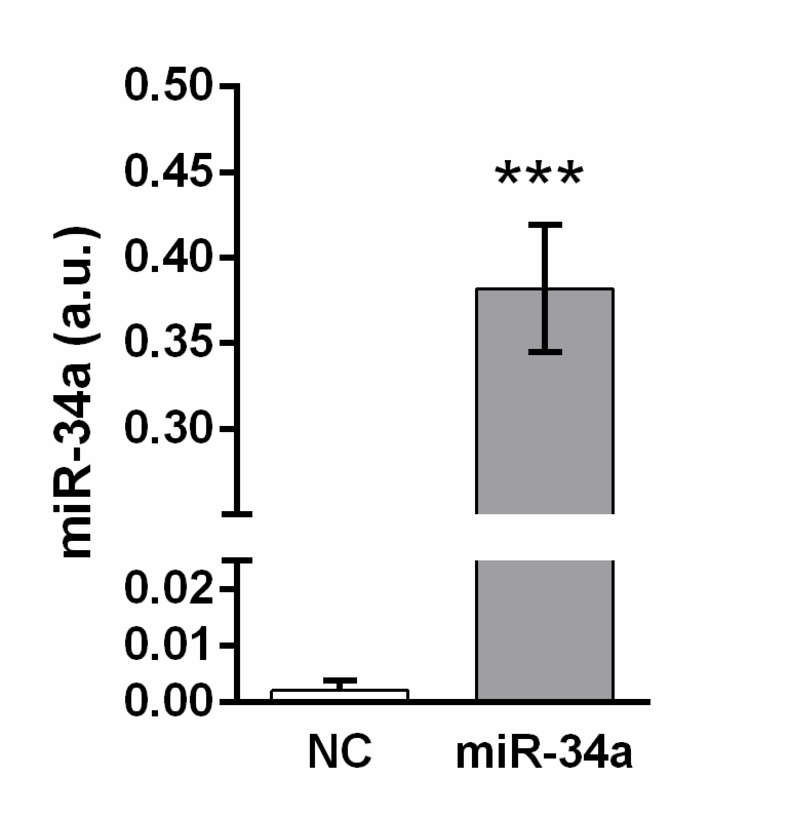

Supplement: S1 Fig — Results are displayed as mean ± SEM (n = 5–6). Statistical differences were determined by a Student’s unpaired t-test (***p < 0.001). NC–negative control. (TIF) [file pone.0292880.s001.tif]

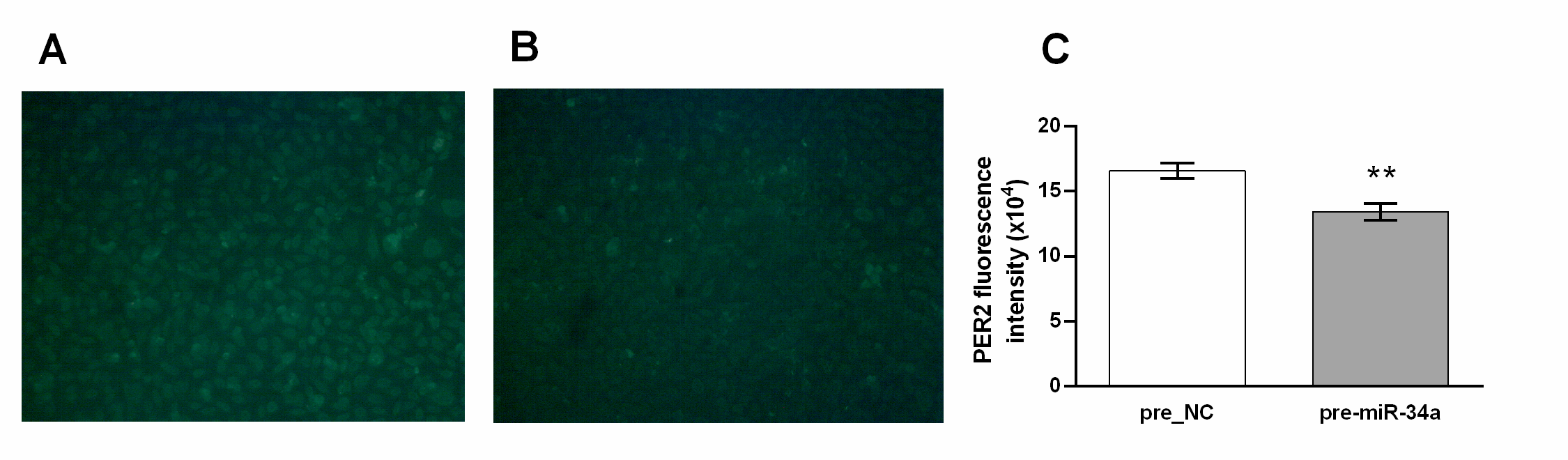

Supplement: S2 Fig — Fluorescent staining of PER2 protein in control (A) and pre-miR-34a-transfected DLD1 cells (B). Fluorescence intensity (C) is expressed as mean ± SEM (n = 12). Images were taken with an inverted fluorescence microscope with a magnification of 100x. Statistical differences were determined by an unpaired Student‘s t-test (*p < 0.05). preNC–negative control. (TIF) [file pone.0292880.s002.tif]
