# Supplementary material for: Effect of miR-34a on the expression of clock and clock-controlled genes in DLD1 and Lovo human cancer cells with different backgrounds with respect to p53 functionality and 17β-estradiol-mediated regulation
Source: PLoS One. 2023 Oct 13;18(10):e0292880. doi: 10.1371/journal.pone.0292880 (PMC10575541; doi:10.1371/journal.pone.0292880)
Supplement: S1 Table — (DOC) [file pone.0292880.s003.doc]

**S1 Table. Sequences of the primers used in real-time polymerase chain reaction and reverse transcription**

| **Gene** | **Strand** | **Sequence** | **Accession number** |
| --- | --- | --- | --- |
| *per2* | Forward | 5′-AATGCCGATATGTTTGCGGT-3′ | NM_022817.1 |
|  | Reverse | 5′-GCATCGCTGAAGGCATCTCT-3′ |  |
| *clock* | Forward | 5′-ATCACAGGGCACCACCCA-3′ | NM_001267843.1 |
|  | Reverse | 5′- TGAGTCTGAAGCCAAATCCAC-3′ |  |
| *bmal1* | Forward | 5′-ACTTCCCCTCTACCTGCTCAA-3′ | NM_001297724.1 |
|  | Reverse | 5′-TGTCTTCATCCAGCCCCATC-3′ |  |
| *sirt1* | Forward | 5′-CCGGAAACAATACCTCCACC-3′ | NM_001142498.2 |
|  | Reverse | 5′-CACATGAAACAGACACCCCA-3′ |  |
| *rev-erb* | Forward | 5′-CAAGGCTGTCCCACCTACTTC-3′ | NM_021724.5 |
|  | Reverse | 5′-CCATTCAGCTTGGTGATGTTGC-3′ |  |
| *cyclinD1* | Forward | 5′-CAATGACCCCGCACGATTTC-3′ | NM_053056.2 |
|  | Reverse | 5′-CATGGAGGGCGGATTGGAA-3′ |  |
| *esr1* | Forward | 5′-AGCTTCGATGATGGGCTTAC-3′ | NM_000125.4 |
|  | Reverse | 5′-TTTTCCCTGGTTCCTGTCCAA-3′ |  |
| *esr2* | Forward | 5′-TGAGGGGAAATGCGTAGAAGG-3′ | NM_001437.2 |
|  | Reverse | 5′-CGTTCAGCAAGTGAGCCAG-3′ |  |
| *gper1* | Forward | 5′-GGTCTCTTCCTCTCTCTAGCCCT-3′ | NM_001098201.3 |
|  | Reverse | 5′-CTCCTTGGTCAGGCCCATCAG-3′ |  |
| *u6* | Forward | 5′-GCTTCGGCAGCACATATACTAA-3′ | NR_004394.1 |
|  | Reverse | 5′-CGTTCAGCAAGTGAGCCAG-3′ |  |
| *rplp13a* | Forward | 5′-GGACCGTGCGAGGTATGCT-3′ | NM_012423.3 |
|  | Reverse | 5′-ATGCCGTCAAACACCTTGAGA-3′ |  |
| *β-actin* | Forward | 5′-GGACTTCGAGCAAGAGATGG-3′ | NM_001101.5 |
|  | Reverse | 5′-GACTCCATGCCCAGGAAGG-3′ |  |
| *miR-34a-5p* | Forward | 5′-GCAGTGGCAGTGTCTTAG-3′ | MIMAT0000255 |
|  | Reverse | 5′-GGTCCAGTTTTTTTTTTTTTTTACAAC-3′ |  |
| *miRNAs* | Primer for reverse transcription | 5´-CAGGTCCAGTTTTTTTTTTTTTTTVN-3´ | MIMAT0000255 |
