# Supplementary material for: Effect of miR-34a on the expression of clock and clock-controlled genes in DLD1 and Lovo human cancer cells with different backgrounds with respect to p53 functionality and 17β-estradiol-mediated regulation
Source: PLoS One. 2023 Oct 13;18(10):e0292880. doi: 10.1371/journal.pone.0292880 (PMC10575541; doi:10.1371/journal.pone.0292880)
Supplement: Attachment — Submitted filename: Response to Reviewers RM 2023 revision 2 def.doc [file pone.0292880.s004.doc]

**Comenius University Bratislava**
Faculty of Natural Sciences
Department of Animal Physiology and Ethology

Mlynská dolina B-2, 842 15 Bratislava, Slovak Republic

Tel.: 00 421 2/9014 9572

e-mail: herichova1@uniba.sk

https://fns.uniba.sk/kzf/

Ilkovičova 6

842 15 Bratislava 4

________________________________________________________________________________

# *Bratislava, 29^th^ September, 2023*

**Response to reviewers**

**Comments from the editorial board**

Please review your reference list to ensure that it is complete and correct. If you have cited papers that have been retracted, please include the rationale for doing so in the manuscript text, or remove these references and replace them with relevant current references. Any changes to the reference list should be mentioned in the rebuttal letter that accompanies your revised manuscript. If you need to cite a retracted article, indicate the article’s retracted status in the References list and also include a citation and full reference for the retraction notice.
 - We did our best to avoid retracted articles. We are not aware of any retracted article in our list of references. All cited articles were checked in the database <http://retractiondatabase.org/RetractionSearch.aspx>

**Comments and Suggestions for Authors**

**Reviewer #1:**

Dear reviewer,

Thank you very much for your valuable time, suggestions, constructive comments, advice and questions; we appreciate them very much. All your suggestions and comments were implemented into the text, and questions were answered; please see the detailed responses bellow. We hope that you will find the revised MS acceptable for publication.

With many thanks and best regards,

The authors

Reviewer #1: Dear authors,

there are still many correction to do, specially in the discussion.

Line 373: when you refer to another work, you should write "has been" instead od "was".
Line 380;potentiate THE effects of each other.
Line 384: As under THE present experimental conditions miR-34a induced a decrease in per2 expression in DLD1 and....... I canceled "the"
line 403: The following sentence is not clear.
If you did the analysis in only DLD1 cells, how can you state that this effect is independent of the p53 status? "In our study administration of miR-34a significantly inhibited cell proliferation measured by an MTS assay without regard to the mutated form of p53 in DLD1 cells."
Line 413: It was shown that silencing of sirt1.......if this stament is refered in ref 28, you should say" It HAS been shown......
413 upregulates and overexpression of sirt1 causes a decrease in per2 expression.Line 418:r"recent experimental condition " change recent for i"n the present experimental conditions"
Line 433:"It seems that administration of ectopic miR-34a does not induce wound closure directly but via the MDM4/p53 pathway" why?
Line 435: You should not begin a paraghraph with "Morover" Just continue in the previous line.
Line 446: change In recent study miR-34a inhibited expression" for " In the present study"
Line 455: "A recent study supports" Do you mean " In the present study"
Line 463: In a recent study " change to " In the present study"
Line 464 It should be: "In spite of that E2-induced cyclin D1,..."
Line 467:We suppose that induction of cyclin D1 expression was mediated by SIGNALING OF MINORIT esr1 receptors present in DLD1 cells [13,107].
Line 489:Among major limitations of THE PRESENT study Line 509:seems to BE independent of miR-34a
Apart from grammatical errors there are some things that are not very clear.
- Thank you very much for the comments. All suggestions were accepted, and the text was corrected accordingly. The sentence ‘It seems that administration of ectopic miR-34a does not induce wound closure directly but via the MDM4/p53 pathway’ was omitted from the text.

As we are not native English speakers, there will always be some imperfections in the text if write it without professional help. For this reason, we always order the services of a professional agency. Several departments of our university are more than satisfied with the editing performed by Proof-Reading-Service.com (PRS); please see the link below.

<https://www.proof-reading-service.com/about-us/>

This is company is located in England close to the city of Cambridge, and it ranked among the top 10 Online Proofreading Services of the year 2023.

<https://www.papertrue.com/blog/the-top-10-online-proofreading-services/>

The MS was edited by PRS before submission, and we have had it proofread a second time for grammar and sentence structure. We hope that the quality of the text is now sufficient for publication in the prominent and reputable journal PLOS ONE. Please see also the newly released certificate of editing.

In the first paragraph you state that the expression of bmal1 and rev-erba are influenced by miR-34a independent of p53 status but the first is just affected in LoVo cells and the second one, just in DLD1 cells; this should be commented.
Also cyclin D1 is different in LDL1 and LoVo cells
- Thank you for the comment. In the first paragraph of the discussion, we intended to deliver the major idea of the article, that the circadian oscillator can be influenced by miR-34a-5p without respect to p53 functionality. We do not claim that p53 cannot influence the circadian oscillator at all if it is in its unmutated form. However, if p53 is not working, miR-34a-5p can still modify clock gene expression. In the revised version, we went into more detail and added information about particular genes whose expression was influenced in both cell lines. We also specified the cell lines with respect to particular genes.

The statement that miR-34a can influence circadian oscillator functioning also issued from other papers and conference contributions of our group:

Olejárová S, Moravčík R, Herichová I. 2.4 GHz Electromagnetic Field Influences the Response of the Circadian Oscillator in the Colorectal Cancer Cell Line DLD1 to miR-34a-Mediated Regulation. Int J Mol Sci. 2022; 23: 13210. doi:10.3390/ijms232113210

Herichová I, Reis R, Hasáková K, Moravčík R, Vicián M, Zeman M: Sex dependent expression of clock gene in human colorectal cancer tissue. In: Proceeding of 96. Physiological days, Jessenius Faculty of Medicine in Martin UK, Martin, s. 27, 2020.

Jendrisková S, Herichová I: Effect of electromagnetic field and miR-34a-5p on migration and gene expression of colorectal cancer cell line DLD1. In: : Proceeding of 97. Physiological days, Institute of Physiology of the 1. faculty of medicine CU, Charles University, Praha, s. 42, 2022.

That E2 did not influence expression of miR-34a and miR-34a administration did not influence expression of esr2 in DLD1 cells, does not mean that they cannot have a sinergetic effect upon other target genes investigated. From your results, you can only say that there is no sinergic effects of miR-34a and E2 in proliferation and migration of LDL1 cells.

- Thank you for the comment. The sentence ‘E2 co-administration did not modulate the effects of miR-34a, therefore, for now we cannot support our hypothesis that differences observed...’

was changed to:

‘E2 co-administration did not modulate the effect of miR-34a on DLD1 proliferation, so for now we cannot support our hypothesis that differences observed... ’

Our statement that miR-34a and E2 do not act synergically is also based on the observation that while miR-34a inhibited the expression of cyclin D1, administration of E2 had the opposite effect.

Material and Methods
How many independent experiments did you performed for each analysis to get the results? This should be clarified.
- The sentence ‘Experiments were performed three times.’ was included in the section ‘Material and methods’.

Can you please clarify how did you determine the concentation of miR-34a? In my experience, by nanodrop, you can just see total RNA and suppose that if RNA is of good quality, also miRs will be OK.Which quantity of total RNA you used for the experiments.

- Thank you for the comment. We are sorry that the sentence ‘*Micro RNA from the DLD1 cell line was isolated using RNAzol RT (Molecular Research Center). The miRNA concentration and purity were determined using a Simply Nano spectrometer (Ge Healthcare, USA).*’ was unclear and probably led to the impression that the concentration of miR-34a was measured by nanodrop. As stated in the ‘Materials and methods’ section, we used RNAzol RT (Molecular Research Center) to isolate RNA. In particular, we used the ‘Protocol for isolation of large RNA and small RNA fraction’ that allows the separation of long and short RNA molecules; please see the link below.

<https://www.mrcgene.com/wp-content/uploads/2017/04/RNAzolRTMarch2017.pdf>

According to this protocol, it is recommended to measure the concentration of miRNAs similarly to the concentration of long RNA molecules, only that the 260/280 ratio is different.

To avoid misunderstanding concerning miR-34a measurement, we used plural when describing miRNA isolation and made some other minor changes. We also included information about the particular protocol that was used for miRNA RT.

We did not use total RNA in reverse transcription. To synthesize cDNA from mRNA, we used 0.3 ug of the RNA fraction with long molecules, which is well within the range (1 pg–1 μg) of the kit mentioned in the section ‘Materials and methods’; please see link bellow.

<https://worldwide.promega.com/-/media/files/resources/protocols/technical-manuals/0/improm-ii-reverse-transcription-system-protocol.pdf?rev=5d3ab0d332a543b9847784041cdcf152&sc_lang=en>

The concentration of miR-34a was measured by real-time PCR using relative quantification. After miRNA isolation, sequences were extended by adenylation and adding a tag during reverse transcription. This was followed by a PCR reaction with two primers that allowed specific measurement of miR-34a-5p from pool of all isolated and transcribed miRNAs. The majority of the miRNA sequence is used for priming by the forward primer. The specificity of the reverse primer is based on partial overlap with the miRNA sequence. This technique is described in the paper (Barcells et al., 2011; 197 references in WOS) and has been adapted for commercial use by Qiagen (please see link below).

<https://www.qiagen.com/us/products/discovery-and-translational-research/pcr-qpcr-dpcr/qpcr-assays-and-instruments/mirna-qpcr-assay-and-panels/mircury-lna-mirna-pcr-assays>

The sequences of all primers are provided in supplementary table 1. This method of miR-34a-5p measurement has been used previously in the paper Hasakova et al. (2019). These details are now provided in the section ‘Materials and methods’.

Balcells I, Cirera S, Busk PK. Specific and sensitive quantitative RT-PCR of miRNAs with DNA primers. BMC Biotechnol. 2011; 11:70. doi: 10.1186/1472-6750-11-70.

Hasakova K, Reis R, Vician M, Zeman M, Herichova I. Expression of miR-34a-5p is up-regulated in human colorectal cancer and correlates with survival and clock gene PER2 expression. PLoS One. 2019; 14(10):e0224396. doi: 10.1371/journal.pone.0224396.
